# A national cross-sectional study on nurses' intent to leave and job satisfaction in Lebanon: implications for policy and practice

**DOI:** 10.1186/1472-6955-8-3

**Published:** 2009-03-12

**Authors:** Fadi El-Jardali, Hani Dimassi, Nuhad Dumit, Diana Jamal, Gladys Mouro

**Affiliations:** 1 Department of Health Management and Policy, Faculty of Health Sciences, American University of Beirut, Beirut, Lebanon; 2 School of Pharmacy, Lebanese American University, Beirut, Lebanon; 3 School of Nursing, Faculty of Medicine, American University of Beirut, Beirut, Lebanon; 4 Department of Health Management and Policy, Faculty of Health Sciences, American University of Beirut, Beirut, Lebanon; 5 American University of Beirut Medical Center, Beirut, Lebanon

## Abstract

**Background:**

Lebanon is perceived to be suffering from excessive nurse migration, low job satisfaction, poor retention and high turnover. Little is known about the magnitude of nurse migration and predictors of intent to leave. The objective of this study is to determine the extent of nurses' intent to leave and examine the impact of job satisfaction on intent to leave. Intent to leave was explored to differentiate between nurses who intend to leave their current hospital and those intending to leave the country.

**Methods:**

A cross-sectional design was used to survey nurses currently practicing in Lebanese hospitals. A total of 1,793 nurses employed in 69 hospitals were surveyed. Questions included those relating to demographic characteristics, intent to leave, and the McCloskey Mueller Satisfaction Scale. Univariate descriptive statistics were conducted on sample's demographic characteristics including gender, age, marital status and educational level. Bivariate associations between intent to leave and demographic characteristics were tested using Pearson Chi-square. Differences in satisfaction scores between nurses with and without intent to leave were tested using t-test and ANOVA f-test. A multinomial logistic regression model was created to predict intent to leave the hospital and intent to leave the country.

**Results:**

An alarming 67.5% reported intent to leave within the next 1 to 3 years, many of whom disclosed intent to leave the country (36.7%). Within nurses who reported an intent to leave the hospital but stay in Lebanon, 22.1% plan to move to a different health organization in Lebanon, 29.4% plan to leave the profession and 48.5% had other plans. Nurses reported being least satisfied with extrinsic rewards. A common predictor of intent to leave the hospital and the country was dissatisfaction with extrinsic rewards. Other predictors of intent to leave (country or hospital) included age, gender, marital status, degree type, and dissatisfaction with scheduling, interaction opportunities, and control and responsibility.

**Conclusion:**

Study findings demonstrate linkages between job satisfaction, intent to leave, and migration in a country suffering from a nursing shortage. Findings can be used by health care managers and policy makers in managing job satisfaction, intent to leave and nurse migration.

## Background

Lebanon has the 8^th ^lowest nurse density in the Eastern Mediterranean Region (EMR) and is believed to be suffering from excessive nurse migration, low job satisfaction, poor retention and high turnover [1-3]. Yet, little is known on why Lebanese nurses leave. This paper examines the perceived intent to leave of Lebanese nurses as it relates to job satisfaction. More specifically, this study examines the difference between nurses' intent to leave the hospital where they work and their intent to leave the country and the factors associated with them, particularly as it relates to job satisfaction. Intent to leave in this study is defined as nurses' intent to potentially quit their current job to pursue other opportunities. The findings of this study can provide some insight to Lebanese health care managers, professional associations and policy makers in addition to managers and policy makers in other countries in the EMR in an effort to better retain their nursing workforce.

Evidence shows a relationship between nursing shortage and low job satisfaction; a relationship between low job satisfaction and intent to leave [4]; and a relationship between low job satisfaction and nurse migration [5]. While existing studies generally focus on one concept or on the relationship between two concepts, no documented studies explore the relationship between nurses' job satisfaction, intent to leave and migration, particularly in countries suffering from nursing shortages. There is also no conceptual framework that relates those concepts together. While this paper does not propose such a framework, it does suggest a possible association between nurses' job satisfaction, intent to leave and migration in a country suffering from a nursing shortage.

The purpose of this study is to examine the relationship between nurses' job satisfaction and intent to leave. The study data were based on a cross-sectional survey of 1,793 nurses employed in 69 hospitals in Lebanon. In the analysis, the dependant variable, intent to leave, was split into three groups: those who intend to leave the hospital, those who intend to leave the country (migrate), and those who intend to stay in their current job. Independent variables included nurses' demographic characteristics and job satisfaction.

## Study rationale

Lebanon, like many other countries in the region, is struggling with a shortage of qualified nurses. Migration of professionals is not uncommon in Lebanon where a culture of migration pervades [6]. Lebanon has the 8th lowest nurse density in the EMR; it exports nurses to some of the Gulf countries which often engage in active recruitment of Lebanese and other nurses. Local reporting sources indicate that Lebanon is facing a severe shortage of nurses compared to physicians, with an estimated ratio of 1 nurse for every 1600 patients in comparison to 1 doctor for every 170 patients [7]. The 2006 World Health Report indicated a nurse density of 1.18 per 1000 in Lebanon compared to an EMR average of 2.20 per 1000 and a global average of 4.06 per 1000. The report also reported a physician density of 3.25 per 1000 compared to a regional average of 1.14 per 1000 and a global average of 1.70 per 1000 [8].

The status of nurses in Lebanon is not very clear and no official reporting mechanism exists to date. Moreover, little is known about the supply and distribution of nurses in Lebanon due to the lack of a proper surveillance system from which accurate judgments can be derived. Data from the Lebanese Order of Nurses report that a total of 6,026 nurses are ascribed in their database; but an estimated 2,000 additional nurses have yet to register. The majority of nurses (85.3%) are females and 51.5% are below 30 years of age, (mean age is 32.1 ± 8.9) [9]. This is of particular importance since evidence shows that nurses in this younger age group are more likely to leave the profession or migrate [10]. Over 90% of nurses are employed and the majority (75%) holds either a Bachelors' of Science (BSN) or a Technique Superior (TS) (46.4% and 28.6% respectively). There is also a geographic mal-distribution of nurses since the majority is working in urban areas such as Beirut (35.8%) and Mount Lebanon (25.7%) [9]. It should be noted that Lebanon is divided into six administrative regions. These include: Beirut which is home to the country's capital and center of economy and trade, Mount Lebanon which is close in proximity to Beirut, the North which extends towards the Syrian Border, the Bekaa valley which is the country's agricultural hub, the South and Nabatieh which extend farthest south of Beirut [7]. While there is no clear cut definition of urban and rural areas in Lebanon, most of the urban areas in the country are concentrated in Beirut and Mount Lebanon while the remaining regions are composed of rural areas in general but also contain a few major cities.

In terms of nurse migration, data from the Order of Nurses indicate that less than 2% of the Lebanese nurses are working abroad. However, this number should be interpreted with caution since many nurses may have migrated before the establishment of the Lebanese Order of Nurses in 2002 and may not have registered. Moreover, according to the Order, nurses do not regularly update their personal information. Therefore, many nurses who are apparently registered as employed in Lebanon may in fact be working abroad [9]. This notion is supported by a recent study which found that 1 in 5 Lebanese nurses migrate a few years after receiving their BSN [2]. This may also indicate that the previous figure reported by the Order of Nurses may be a gross underestimation; however, an accurate estimate is still lacking. Lebanese nurses' reasons for leaving cited by migrant nurses included the lack of career development opportunities, followed by poor salaries, inequality with other health professionals, and being underestimated as valued health professionals. Furthermore, a prominent nurse recruitment agency surveyed in this study reported that the most frequent reason for leaving indicated by their clients was the search for a better paying position. Migrant nurses reported that a combination of financial and non-financial incentives would encourage them to return to practice in Lebanon. The most recurring incentives to encourage nurses to return to practice in Lebanon included educational support, managerial support, better working conditions, utilization of best nursing practices and autonomy in their practice [2].

While some evidence has been documented on nurses' intent to stay or leave in developed countries, an exhaustive review of the literature revealed that no evidence exists in the EMR, and more specifically in Lebanon. There is a dearth of studies that investigate whether predictors of intent to leave in the region are different from such factors in other regions. In fact, no national study to assess Lebanese nurses' intent to leave and job satisfaction has been done to date. To our knowledge, this is the first study to address this topic in Lebanon and the EMR. The challenges facing the nursing profession in Lebanon brought issues of intent to leave and retention to the attention of the health care managers and the policy makers. These issues provided the background, incentive and guidance to our study.

## Literature on concepts related to intent to leave

### Shortages

The issue of Human Resources for Health (HRH) started to gain more international attention following the 2006 World Health Report. In this report, the World Health Organization (WHO) reported compelling figures about the international shortages in HRH, specifically nurses and particularly in developing nations. In their report, the WHO recommended that countries give high priority to developing effective workforce strategies that focus on three core elements: improving recruitment, helping the existing workforce perform better, and slowing the rate at which health workers leave the workforce [8]. The Kampala declaration in 2008 also stressed the importance of developing strategies to combat shortages and the need for effective recruitment and retention approaches to combat shortages and improve retention [11].

The world is currently witnessing a critical shortage of qualified and well-trained nurses to meet the changing health needs of its growing population [12,13]. While this shortage may vary in terms of size and severity across developed and developing nations, its impact is heavier on developing countries. In fact, the burden of the shortage weighs more heavily on Low and Middle Income Countries (LMICs) as they are dealing with poor health indicators, and the lack of reliable and complete information on the number and qualifications of their health workforce, including nurses. Recent evidence shows that shortages are a symptom of inadequate policies on recruitment and retention of health workers [14].

The nursing shortage is multifactorial and has resulted from a combination of factors. Nursing shortage is an outcome of increasing demand for nurses, declining nursing school enrolment, better job opportunities for nurses in other countries, and aging workforce among many others [15,16]. This shortage coincides with increasing demand for healthcare, longer life expectancy, and an increase in rates of chronic diseases [17]. Nursing shortage is a critical challenge since evidence strongly suggests that the availability of sufficient and properly trained health workers can save lives [8]. A recent study found that increasing nurse density is significantly associated with lower maternal mortality rates [18]. This is in concordance with a study conducted by Aiken and colleagues [19] that found that an additional nurse per day reduced risk of patient death and also improved patient satisfaction. Another study reported an agreement by physicians that quality of care is compromised by a shortage of nurses [20].

### Job Satisfaction and Intent to Leave

Shortages can be a symptom of low job satisfaction, poor management and lack of organizational support [14]. Shortages are resulting in heavy workload which is a precursor to job stress and burnout which have also been linked to low job satisfaction. Nurses' job satisfaction is an elusive concept which is defined within its extrinsic and intrinsic values [21]. Extrinsic values encompass the tangible aspects of the job including wages, benefits and bonuses, whereas intrinsic values include status, recognition, personal and professional development opportunities, and other similar factors [21]. Reasons for nurse dissatisfaction have been well documented in the nursing literature. Such reasons include lack of involvement in decision making, poor relationship with management, low salaries and poor benefits, lack of job security, poor recognition and lack of flexibility in scheduling [22]. Nurse dissatisfaction has been also linked to emotional exhaustion and burnout which can affect patient outcomes [23].

Job dissatisfaction is a primary predictor of nurses' intent to leave (quit their current job) [4,24,25]. A study conducted in the United States presented evidence showing that dissatisfied nurses were 65% more likely to have an intent to leave compared to their satisfied counterparts [4]. Other predictors of intent to leave vary from low salaries and fringe benefits, inflexible work schedule [17,24,26], career advancement prospects [24,27], in addition to poor management and job stress [27]. Nurses' intent to leave has also been linked to demographic characteristics such as age and gender [26], situational factors such as family obligations, early retirement [27], and length of service [26]; low levels of motivation, emotional exhaustion and burnout; and to the poor social image of the nursing profession [24]. It is worth noting that job satisfaction has also been found to be a better predictor of intent to leave as compared to the availability of other employment opportunities [4]. However, no research evidence has been documented in nursing literature on the link between intent to leave and nurse migration.

### Nurse Migration

Nursing has been described as a mobile profession. Nurses traditionally migrated in search for professional development that is typically not attainable in their home countries. Literature showed that nurses tend to migrate to seek better wages, better working conditions, better living standards and personal safety [15]. Reasons for out-migration, also known as "push" factors, predominantly include poor working environment, heavy workloads, lack of flexibility in scheduling, promotion opportunities, work incentives as well as remuneration [5]. Economic instability in some developing countries leads to poorly funded health care systems that in turn impel the exodus of health personnel. These reasons along with lack of professional development drive this movement [28] in that they induce vulnerability in the health workforce. This allows developed countries to capitalize on such instability and in turn provide desirable "pull factors" such as better wages and working conditions, as well as opportunities for advancement and benefits [28]. The effect of migration is most highly pronounced in donor countries due to the loss of skilled personnel in addition to the loss of economic investment in nursing education [15]. In contrast, recipient countries are at an advantage since they are receiving skilled nurses who can enter the workforce with the appropriate preparation and training [15].

### Knowledge Gap

As shown in the literature review, studies generally focus on one concept such as job satisfaction or shortages, or on the relationship between two concepts, i.e. job satisfaction and intent to leave or migration. Yet, studies addressing the relationship between nurses' job satisfaction, intent to leave and migration, particularly in the context of a country with a shortage of nurses were not found. Studies on nurses' intent to leave do not clearly specify whether nurses simply want to leave the hospital where they work or if they intend to leave their country. Furthermore, there is no conceptual framework yet that relates those concepts together. Some researchers presented frameworks related to migration relating to issues other than job satisfaction. For instance, Lee [29] proposed several determinants and predisposing factors that facilitate migration, but not specifically for nurses. The decision to migrate is mostly triggered by economic incentives but can also be determined by distance, the destination country (inhabitants, language, quality of life), and migration streams [29]. Akl and colleagues [6], on the other hand, proposed a framework related to medical student's decision to train abroad which is based on the push and pull theory of Lee [29]. Repel and retain factors for Lebanese medical students include personal, social, professional and political factors [6]. Tourangeau and Cranley [13] introduced a framework for understanding the determinants of nurses' intention to remain employed. The authors used this framework as a model that incorporated predictors of intention to remain employed and consequently nurse retention. The predictors included organizational commitment, job satisfaction, burnout, work group cohesion and collaboration, managers' ability and support, personal characteristics and other "unknown factors" [13].

Evidently, a knowledge gap exists when exploring the relationship between nursing shortage, job satisfaction, intent to leave and nurse migration. Although this paper does not develop a conceptual framework, it sheds light on a possible association between these concepts and highlights the need for more relevant research on this topic. In light of the nursing shortage in Lebanon, this study examines how nurse job satisfaction can impact intent to leave the hospital and the country. Nurses' leaving the country is assumed in this study as nurse migration.

The objectives of this study are to determine the extent of nurses' intent to leave in Lebanon and to examine the impact of job satisfaction on intent to leave. In this research, intent to leave was further explored to differentiate between nurses who want to leave the hospital and those who want to leave the country. Furthermore, this inquiry determines the factors associated with nurses' intent to leave (hospital or country) in relation to demographic characteristics and more specifically nurses' job satisfaction.

## Methods

### Study Design

A cross-sectional design was used in this study to survey nurses currently practicing in Lebanese hospitals. The sample included Registered Nurses with at least 1 year of experience and holding a BSN, or Baccalaureate Technique (BT), or TS, or License Technique (LT) and Diploma; all of whom are considered registered nurses [30]. A total of 120 hospitals in Lebanon were contacted for a preliminary assessment. A total of 69 of the 76 hospitals who responded to our initial assessment consented to participate in this survey. Of the sampled hospitals, 47 (61.8%) were small-sized having 20 to 100 beds, 15 (19.7%) were medium-sized having 101–200 beds and 7 (9.2%) were large-sized having more than 200 beds.

### Survey Instrument

The questionnaire (Additional file 1) used for data collection included a section on demographic characteristics of the nurses (gender, age, marital status, etc.), and a section on intent to leave. Nurses were asked whether they plan to leave their job within the next 1 to 3 years and to include their potential plans after leaving. In addition to the sections above, the McCloskey-Mueller Satisfaction Scale (MMSS) was used to assess job satisfaction. This scale is composed of 31-items scored on a 5-point Likert scale (1 for Very Dissatisfied, 2 for Moderately Dissatisfied, 3 for Neutral, 4 for Moderately Satisfied and 5 for Very Satisfied). The 31 MMSS items are classified into 7 different subscales which are; satisfaction with extrinsic rewards; scheduling; balance of family and work; co-workers; interaction opportunities; praise and recognition; and control and responsibility [31].

A panel composed of nursing professionals, including a nursing director, a nursing specialist and a nursing researcher worked with the research team to adapt the questionnaire to better fit the context of Lebanese hospitals. The MMSS was revised and slightly modified, particularly the wording of some questions to better fit the context of Lebanese hospitals. One of the recommendations of the panel was to drop the neutral point of the Likert scale. The panel also recommended changing the wording of some of the items in the MMSS without changing their meaning.

The questionnaire was translated to Arabic and French then back-translated to English and compared to the original. No major differences were found. All language versions were piloted with 15 nurses prior to data collection; these nurses were excluded from the final sample. Pilot participants were asked about the clarity of the questions, the format of the questionnaire, and the clarity of instructions. Since they found the survey to be easy and straightforward, no major changes were made; only minor changes to the wording of few questions were made.

### Sampling and data collection

**Figure 1:**
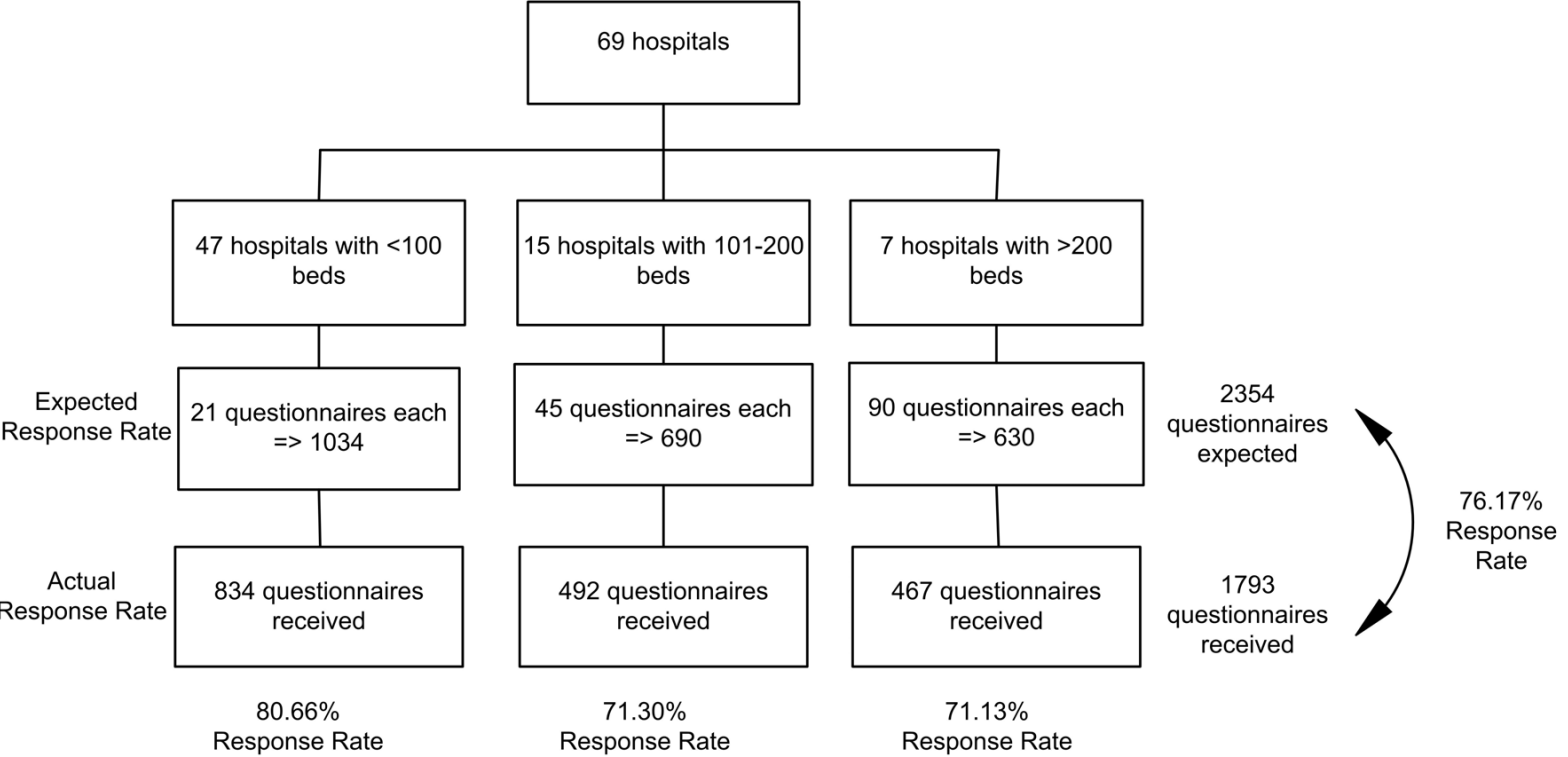
**Sampling and Response Rate**.

Nursing directors at the sampled hospitals were asked to distribute the questionnaires to nurses fitting the afore-mentioned eligibility criteria. In attempt to sample at least 50% of practicing nurses at each hospital, an average of the estimated number of nurses within each size category was computed. Each small-sized hospital (20–100 beds) was asked to return 21 questionnaires, while each medium-sized (101–200 beds) was requested to return 46 questionnaires and each large-sized hospitals (>200 beds) was asked to return 90 questionnaires. A total of 2,354 questionnaires were expected to be filled. A total of 1,793 questionnaires were collected resulting in an overall 76.17% response rate (See Figure 1).

Ethical approval for this study was obtained from the university's research board before initiation of the study. The questionnaire had an introductory section explaining the purpose of the study, the freedom of choice to fill the questionnaire, and assurance of anonymity of respondents.

### Data Management and Analysis

After completing data collection, a random sample of 10% of the questionnaires was checked for accuracy and completeness to ensure quality and integrity of the collected data. A data entry interface was developed using CSPro 3.2 to minimize data entry errors. After auditing 20% of entered questionnaires to check on the quality of data entry, data were exported to the Statistical Package for Social Science (SPSS), version 16.0. All analyses were carried at the 0.05 significance level.

The MMSS scores were calculated for each of the 7 subscales. Scores were created by summation of the items within the scales and dividing by the number of items with non-missing values. This produced a score that varies between 1 and 4 for each subscale with higher scores indicating higher satisfaction. To test the reliability of the scale, Alpha Cronbach was computed for each of the subscales. All values were found to be acceptable and are reported in Table 1.

**Table 1:** Comparison of means of MMSS subscales against intent to leave*

					**Intent to Leave Job**		
					
**Satisfaction with...**	**Items**	**Alpha Cronbach**	**Overall Mean (SD)**	**Stay Mean (SD)**	**Total Mean (SD)**	**Leave Hospital Mean (SD)**	**Leave Country Mean (SD)**
Co-workers^(a, b, c)^	2	0.640	3.17 (0.53)	3.26 (0.51)	3.13 (0.54)	3.13 (0.54)	3.14 (0.54)
Interaction opportunities^(a, b, c)^	4	0.774	2.90 (0.50)	2.98 (0.50)	2.87 (0.49)	2.87 (0.47)	2.86 (0.51)
Praise and recognition^(a, b, c)^	4	0.755	2.81 (0.56)	2.95 (0.49)	2.74 (0.57)	2.76 (0.55)	2.70 (0.60)
Control & Responsibility^(a, b, c, d)^	5	0.787	2.71 (0.52)	2.85 (0.48)	2.65 (0.52)	2.68 (0.51)	2.59 (0.52)
Scheduling^(a, b, c)^	6	0.832	2.66 (0.49)	2.77 (0.48)	2.61 (0.48)	2.61 (0.46)	2.60 (0.51)
Professional opportunities^(a, b, c, d)^	4	0.812	2.50 (0.51)	2.60 (0.48)	2.45 (0.51)	2.48 (0.50)	2.41 (0.53)
Balance between family and work life^(a, b)^	3	0.436	2.39 (0.40)	2.43 (0.37)	2.37 (0.42)	2.36 (0.43)	2.39 (0.39)
Extrinsic rewards^(a, b, c)^	3	0.658	2.29 (0.61)	2.44 (0.62)	2.21 (0.60)	2.21 (0.60)	2.21 (0.59)

***Overall satisfaction***^*(a, b, c)*^	***31***	***0.924***	***2.62 (0.23)***	***2.65 (0.23)***	***2.61 (0.23)***	***2.61 (0.53)***	***2.60 (0.23)***

* Scores are on a scale from 1 to 4 in the McLoskey Mueller Satisfaction Scale

a- Significant difference between nurses who want to stay and those who want to leave job (i.e. total)

b- Significant difference between nurses who want to stay and those who want to leave hospital

c- Significant difference between nurses who want to stay and those who want to leave country

d- Significant difference between nurses who want to leave the country and those who want to leave hospital

To better understand the differences between nurses who intend to leave the hospital and those who want to leave the country, nurses were divided into three groups: those who intend to leave the hospital, those who intend to leave the country and those who want to stay in their current job.

Univariate descriptive statistics were conducted to summarize the demographic characteristics of the sample including gender, age, marital status and educational level. The proportions of nurses who intend to leave (total, hospital and country) were calculated along with a 95% Confidence Interval (CI). At a second stage, bivariate associations between intent to leave and demographic characteristics were tested using Pearson Chi-square. Differences in satisfaction scores between nurses with and without an intent to leave were tested using t-test and ANOVA f-test with multiple comparison corrected using the Bonferroni method. Finally, multinomial logistic regression model was created to predict the intent to leave the hospital and intent to leave the country. The dependant variable in this analysis was intent to leave (hospital or country) whereas the independent variables included gender, age, degree type, region, and dissatisfaction on items in MMSS subscales. The coefficients produced by the model were exponentiated to produce Odds Ratios (OR) and standard errors were used to calculate the 95% CI.

## Results

### Demographics

As detailed in Table 2, the majority of nurses that participated in the study were females (80.9%), aged less than 30 (62.9%) and never married (57.1%). When examining the distribution across the different regions in Lebanon, over 40% of the sampled nurses were found to reside in Beirut and Mount Lebanon (16.8% and 27.3% respectively) while less than 30% resided in the North, Bekaa, South and Nabatieh (See Table 2). A total of 34.8% of the nurses held a university degree while 64% held a technical degree. Specifically, a little over 30% of the sampled nurses held a BSN in nursing, 22% held a TS, 14.2% held a BT, 13.4% an LT, and approximately 12% had a nursing diploma (See Table 2).

**Table 2:** Demographic profile of sampled nurses

	**N (%)**
**Age**	
Below 30 years	1127 (62.9%)
Between 30 and 45 years	560 (31.2%)
Between 46 and 55 years	58 (3.2%)
Over 55 years	7 (0.4%)
Missing	41 (2.3%)

**Gender**	
Female	1450 (80.9%)
Male	335 (18.7%)
Missing	8 (0.4%)

**Marital Status**	
Never married	1023 (57.1%)
Ever Married	752 (41.9%)
Missing	18 (1.0%)

**Region**	
Beirut	302 (16.8%)
Mount Lebanon	490 (27.3%)
North	166 (9.3%)
Bekaa	158 (8.8%)
South	140 (7.8%)
Nabatieh	71 (4.0%)
Missing	466 (26.0%)

**Degree Type**	
University	624 (34.8%)
*BSN*	*575 (32.1%)*
*Masters*	*49 (2.7%)*
Technical	1148 (64.0%)
*Diploma*	*214 (11.9%)*
*BT*	*254 (14.2%)*
*TS*	*395 (22.0%)*
*LT*	*241 (13.4%)*
*Others*	*55 (3.1%)*
*Missing*	*10 (0.6%)*
Missing degree type	21 (1.2%)

### Intent to Leave

Only 32.5% of the nurses reported an intent to stay in their current job whereas an alarming 67.5% reported an intent to leave within the next 1 to 3 years (See Table 3). When asked to report their plans after leaving, 36.7% disclosed that they plan to leave the country. Within the remaining 63.3% of nurses who reported an intent to leave the hospital but stay in Lebanon, 22.1% plan to move to a different health organization in Lebanon, 29.4% plan to leave the nursing profession while the rest had other plans such as taking care of their children and other dependants, or continuing their education. Approximately 60% of the nurses reporting an intent to leave believed that it would be easy to find another job in nursing and 62.9% indicated that they would not choose nursing again.

**Table 3:** Intent to leave Job, plans after leaving, ease of finding another job and choosing nursing again (N = 1793)

	**N**	**%**	**95% CI**
**Intent to leave current job**			
Intent to leave	1192	67.5%	65.2% – 69.6%
Intent to stay	575	32.5%	30.4% – 34.8%

**Plans after leaving**			
Leave country	437	36.7%	34.0% – 39.4%
Leave hospital, but stay in Lebanon to...	755	63.3%	60.6% – 66.0%
*Move to a different health organization*	*167*	*22.1%*	*19.3% – 25.2%*
*Leave nursing profession*	*222*	*29.4%*	*26.3% – 32.8%*
*Other plans*	*366*	*48.5%*	*44.9% – 52.0%*

**Finding another acceptable job in nursing for nurses with intent to leave would be...**			
Easy	688	59.1	56.3% – 61.9%
Difficult	431	40.9	38.1% – 43.7%

**Given the opportunity to start all over, would nurses intending to leave choose nursing as a profession?**			
Yes	431	37.1	34.3% – 39.9%
No	732	62.9	60.1% – 65.7%

### McLoskey Mueller Satisfaction Scale

The analysis of the MMSS scale revealed that nurses were least satisfied with extrinsic rewards (mean = 2.29, SD = 0.61) but most satisfied with co-workers (mean = 3.17, SD = 0.53). Nurses with an intent to leave had consistently lower satisfaction scores on all subscales (See Table 1). This holds true whether nurses want to leave the hospital or leave the country. Upon further comparing nurses who wanted to leave the hospital against those who want to leave the country, statistical significance was observed for two subscales (control and responsibility and professional opportunities) showing that nurses with an intent to leave the country had lower satisfaction scores.

### Correlates with intent to leave

In Additional File 2, bivariate associations of demographic characteristics of nurses and intent to leave are reported at two levels: one with intent to leave as a total and another with intent to leave divided into leaving the hospital and leaving the country. Associations for each of the items are reported individually.

#### Age

With respect to age, those who intend to leave are more likely to be younger than 30 years of age (60.9% vs. 55.5%, p-value < 0.001) and this is more true for nurses who want to leave the country (79.2%, p-value < 0.001) (See Additional File 2).

#### Gender

Nurses with an intent to leave are more likely to be males (20.1% vs. 15.9%, p-value 0.037). However, this finding could be truer for those who intend to leave the country (32.7%) than those who intend to leave the hospital (12.7%, p-value < 0.001).

#### Marital Status

Nurses with an intent to leave the country are much more likely to be never married (72.0%) than those who have an intent to leave the job (52.3%) or have no intent to leave (53.5%, p-value < 0.001) (See Additional File 2).

#### Region

Intent to leave was higher for all regions with the exception of Mount Lebanon where intent to stay (43.7%) was higher than intent to leave (33.8%). However, intent to leave in Mount Lebanon (33.8%) was still higher than Beirut (24.3%) (See Additional File 2). Nevertheless, it should be noted that intent to leave the hospital was higher for North, Bekaa and Nabatieh as compared to intent to leave the country. This may indicate some trends in internal migration; nurses leaving these regions may be looking for employment in urban regions of the country.

#### Degree type

Furthermore, Additional File 2 shows that 44.4% of nurses with an intent to leave the country have a university degree compared to 31.3% for those who want to leave the hospital and 34.1% for those with no intent to leave (p-value < 0.001).

#### Finding another job in nursing

As observed in Additional File 2, 62.5% of nurses with an intent to leave the country believe it is easy to find another job in nursing compared to 57.1% of nurses who want to leave the hospital and 53.6% of nurses without an intent to leave (p-value 0.018). This indicates that regardless of their intent to leave or stay, nurses believe it would be easy to find another acceptable job in nursing, which might ultimately increase likelihood of leaving.

#### Choosing nursing again

While more than half of the nurses (58.4%) who have no intent to leave would choose nursing as a profession again, this proportion was much lower for those who had an intent to leave (37.1%, p-value < 0.001) (See Additional File 2).

### Multinomial Logistic Regression Results

To further explore the association between nurses' demographic characteristics and their degree of job satisfaction as they relate to intent to leave, a regression model was constructed. Multinomial regression analysis revealed interesting observations for nurses who want to leave the hospital and those who want to leave the country. Factors associated with intent to leave varied between nurses who want to leave the hospital and those who want to leave the country.

#### Age

Younger nurses were found to report an intent to leave the country at higher odds than their counterparts (OR = 1.961; this is the inverse of OR = 0.510) (See Table 4).

**Table 4:** Regression Model testing for the predictors of Intent to leave hospital and country against Intent to Stay

	**Leave Hospital OR (CI)**	**P-value**	**Leave Country OR (CI)**	**P-value**
**Age**	0.871 (0.678–1.119)	0.871	0.510 (0.364–0.714)	**<0.001**
**Gender**				
Male	0.666 (0.457–0.972)	**0.035**	2.488 (1.712–3.623)	**<0.001**
Female	1		1	
**Marital Status**				
Never married	0.946 (0.708–1.265)	0.711	1.674 (1.178–2.380)	**0.004**
Ever married	1		1	
**Degree type**				
University	0.938 (0.701–1.255)	0.938	1.421 (1.022–1.977)	**0.037**
Technical	1		1	
**Region**				
Mount Lebanon	0.637 (0.441–0.922)	**0.017**	0.601 (0.398–0.908)	**0.016**
North	0.901 (0.553–1.468)	0.675	0.620 (0.350–1.098)	0.101
Bekaa	0.870 (0.531–1.427)	0.582	0.529 (0.294–0.953)	**0.034**
South	1.491 (0.864–2.573)	0.151	1.041 (0.565–1.917)	0.899
Nabatieh	1.116 (0.575–2.166)	0.746	0.727 (0.335–1.579)	
Beirut	1		1	
**Less satisfaction with**				
Co-workers	3.521 (0.866–1.634)	0.284	0.876 (0.606–1.266)	0.481
Interaction Opportunities	0.693 (0.479–1.001)	**0.051**	0.833 (0.546–1.272)	0.399
Praise	1.344 (0.936–1.927)	0.109	1.493 (0.990–2.252)	0.056
Control and Responsibility	1.163 (0.796–1.701)	0.435	1.695 (1.095–2.625)	**0.018**
Scheduling	1.508 (1.060–2.146)	**0.022**	1.340 (0.900–1.992)	0.746
Professional Opportunities	0.107 (0.760–1.513)	0.691	1.531 (0.696–1.531)	0.873
Extrinsic Rewards	1.595 (1.225–2.075)	**0.001**	1.712 (1.267–2.315)	**<0.001**
Balance between family and work life	1.116 (0.772–1.610)	0.560	0.951 (0.618–1.462)	0.818
P-Value	**<0.001**			
N	1263			
Goodness of Fit				
Pearson	0.338			
Deviance	0.440			

#### Gender

Being a male was found to be a protective factor against intent to leave the hospital (OR = 0.666). Nonetheless, when addressing intent to leave the country, male nurses were found to have 2.488 higher odds.

#### Marital Status

Single nurses were found to have significantly higher odds of intent to leave the country (OR = 1.674, 95% CI = 1.178 – 2.380).

#### Degree Type

University trained nurses were also found to have significantly higher odds of intending to leave the country with an OR of 1.421 (95% CI = 1.022–1.977).

#### Region

Nurses residing in Mount Lebanon were significantly less likely to intend to leave the hospital (OR = 0.637, 95% CI = 0.441–0.922) and leave the country (OR = 0.601, 95% CI = 0.398–0.908). Nurses residing in Bekaa were significantly less likely to intend to leave the country (OR = 0.529, 95% CI = 0.294–0.953).

#### MMSS Scale

Decreased satisfaction with control and responsibility increased odds of intent to leave the country by 1.695 (95% CI = 1.095–2.625). Less satisfaction with scheduling was found to be associated with increased intent to leave the hospital (OR = 1.508, 95% CI = 1.060–2.146). Lower satisfaction with extrinsic rewards was found to be associated with increased intent to leave the hospital (OR = 1.595, 95% CI = 1.225–2.075) and even higher odds of intent to leave the country (OR = 1.712, 95% CI = 1.267–2.315).

## Discussion

The results of this study suggest that a high percentage of nurses intend to leave both the hospital and the country. These figures are alarming given recent evidence that 1 in 5 Lebanese nurse migrate within 1 to 2 years of graduation [18]. Hospitals are also losing nurses to other health organizations; such internal nurse migration can exacerbate the existing geographical maldistribution. A recent assessment of Lebanese hospitals showed that the rate of turnover has increased from 13% in 2004 to almost 17% in 2006 [3]. Although it is clear that Lebanese hospitals are losing their nurses at an increasing rate, there is no evidence of how many nurses are lost to internal migration, how many are lost to external migration and how many are lost to the profession. But any loss, regardless of its destination, is contributing to the overall nursing shortage in Lebanon.

Another significant finding in this study is that 29.4% of nurses with an intent to leave are planning to leave the profession. Nursing has been described as a stepping stone into other careers. Nurses globally are heading towards positions that offer more flexibility and match their professional aspirations. This is in concordance with a study showed by Duffield et al. [32] that showed that nurses believe their education and training offers them a range of skills that can allow them to shift to other employment opportunities. It should be noted that most nurses reporting an intent to leave think it would be easy to find another job in nursing, but would not choose nursing as a profession again. This reflects the unattractive status of the nursing profession in Lebanon. If such issues are not addressed and augmented by effective retention strategies to encourage nurses to remain employed, Lebanon will continue to lose its nurses to other countries but more importantly, to other professions.

Intent to leave and job dissatisfaction have been cited as the best predictors of actual leaving [33,34]. When employees, including nurses, feel more satisfied, they show more commitment to the organization and the profession and have a lower tendency of leaving [35]. The results of Zeytinoglu et al. [35] are in congruence with this study's findings which demonstrate that nurses reporting an intent to stay had higher satisfaction scores than nurses with intent to leave (hospital and country). Furthermore, nurses with an intent to leave country generally had lower satisfaction scores on all MMSS subscales with the exception of those measuring satisfaction with extrinsic rewards and co-workers. Nurses who wanted to leave the country and the hospital were equally dissatisfied with extrinsic rewards. As for the subscale on co-workers, nurses with an intent to leave the hospital were less satisfied than nurses who wanted to leave the country. Thus it is essential that health care managers and policy makers develop a better understanding of the causes of nurse job dissatisfaction and the reasons nurses stay in their jobs. This would help them develop targeted retention strategies to address underlying causes since the socio-demographic characteristics of nurses have been found to affect turnover behavior and also affect the importance of other predictors of turnover [36,37]. For instance, the findings of this study suggest that the profile of nurses who want to stay working in Lebanese hospitals include nurses who are female, aged between 30 and 45 years, married, holders of technical degrees, those who think it would be difficult to find another job and those who would consider choosing nursing as a profession again.

When comparing nurses who intend to leave the hospital to those who intend to leave the country, it was observed that male nurses were more likely to intend to leave the country whereas female nurses were more likely to intend to leave the hospital. Evidence showed that migration of female nurses is increasing [38,39] yet international migration streams are still predominantly composed of males [40]. As for age, nurses younger than 30 years were more likely to intend to leave the country whereas nurses between 30 and 45 years were more likely to intend to leave the hospital. This is in agreement with recent evidence that nurses younger than 30 are more likely to intend to leave [41]. Unmarried nurses were more likely to report an intent to leave the country while married nurses were more likely to intend to leave the hospital. This comes to show that cultural factors in Lebanon may actually have an impact on nurse migration as it is usually not socially acceptable for females to travel and live alone abroad if they are unmarried [6].

University degree nurses were more likely to report an intent to leave the country compared to technical degree nurses who reported an intent to leave the hospital. Recent evidence in Lebanon shows that university trained nurses are in high demand particularly in the oil-rich Gulf countries and other regions [2]. This could be interpreted by the perception that university trained nurses provide superior care [30].

Predictors of nurses' intent to leave the hospital were compared to nurses who intend to leave the country; multinomial regression revealed that less satisfaction with extrinsic rewards increases the odds of intent to leave the hospital and intent to leave the country. Many nurses leave their country in search of professional development opportunities while others leave in search of higher salaries to support their family as many are the sole contributors to total household income [42]. A higher salary was found to be one of the main reasons Lebanese nurses choose to migrate [2]. Furthermore, surveys of nurses showed that higher salaries and better benefits are the most effective recruitment and retention strategies [43]. Health care managers and policy makers should address issues related to salaries, fringe benefits and vacations (taking into consideration the average national income) as they seem to be equally important to nurses with an intent to leave the hospital and country.

Findings in terms of satisfaction with control and responsibility of nurses reveal that it is a predictor of nurses' intent to leave the country. Evidence showed that nurses are more likely to stay in their job if they feel they have sufficient control over their practice, adequate amount of responsibility and autonomy, good collaboration with colleagues and other health professionals and sufficient managerial support [26,44,45]. In addition to those factors, career development, an item within the control and responsibility subscale, was shown to affect decision to migrate [2]. This comes to show that health care managers and policy makers should provide Lebanese nurses with more opportunities for career advancement and also increase their control over their work setting and conditions, increase amount of responsibility and allow them to participate in decision making. This also relates to career advancement opportunities for nurses whereby hospitals can institutionalize career ladder programs that can act as a non-financial incentive and give nurses more control over their career and future plans.

In addition to the extrinsic rewards, two other predictors were found to affect intent to leave the hospital. These include interaction opportunities and scheduling. In fact, interaction with hospital staff, through internal rotation for instance [22,46], is not only cost effective but can also allow the nurse to have a wider range of clinical experience which contributes to career advancement [46]. For the former, health care managers and policy makers should provide Lebanese nurses with more opportunities to interact with other professions and disciplines in addition to opportunities to deliver health care services through multidisciplinary teams.

Since scheduling was shown to impact nurses' decision to leave, nurses require more flexibility and incentives when working day/night shifts, overtime and on weekends. Improving staffing and scheduling conditions is essential in reducing turnover rates at hospitals; this result is ratified by Gulatte et al. [12], Aiken et al. [47]; and Albaugh [22]. Furthermore, Better schedules are particularly important in the context of Lebanon since female married nurses who have kids and other dependants require more flexible schedules that could help them accommodate their family responsibilities [3].

## Conclusion

The findings of this study demonstrate the linkages between job satisfaction, intent to leave, and migration in a country suffering from a nursing shortage; and provide essential background knowledge about the nursing workforce in Lebanon. While this paper does not assume any causation between those concepts, it does suggest some form of an association that requires more research. In the context of Lebanon, it is evident through the findings of this study that low job satisfaction is impacting nurses' intent to leave the hospital and the country which implies migration of nurses within and outside the country. The combined effect of these linkages is exacerbating the existing nursing shortage in Lebanon. The perceived nursing shortage is aggravated by an imbalance between physician and nurse densities, lack of updated data and lack of a national health workforce strategy. Evidence in the literature comes to show that shortages contribute to low job satisfaction which contributes to intent to leave. Given the high intent to leave for respondents in this study, particularly the intent to leave the country, and considering the Lebanese cultural context that encourages migration, there is a high probability that nurses with the intent to leave may eventually migrate out of Lebanon. This makes the nursing workforce problem not only a concern for the individual hospitals, but more of an alarming concern for the heath authorities, specially the Ministry of Health. Therefore, health care managers and policy makers should develop and institutionalize targeted nurse recruitment and retention strategies taking into consideration the predictors and outcomes of nurses' intent to leave both the hospital and country. Rather than having a generic policy that applies to all nurses, the findings of this study suggest that retention policies should be sensitive to the needs and interest of nurses with high risk of leaving.

In terms of regional policy implications, this inquiry established background knowledge since limited information is available on the health workforce in the EMR. The region is particularly facing challenges pertaining to shortages, poor working conditions and remuneration of health professionals, aging workforce, lack of strategies for recruitment and retention, skill and geographical imbalances, absence of HRH databases, lack of a strategy for educational reform and out-migration [18]. The study findings can help inform future decisions that will be taken by health care managers and policy makers particularly in similar Middle Eastern source countries such as Jordan and Egypt. Findings can also help structure future strategies for ministries of health in Lebanon and other similar countries in the region that are struggling with health workforce migration, particularly nurse migration.

## Abbreviations

EMR: Eastern Mediterranean Region; BSN: Bachelors' of Science; TS: Technique Superior; HRH: Human Resources for Health; WHO: World Health Organization; LMIC: Low and Middle Income Country; BT: Baccalaureate Technique; LT: License Technique; MMSS: McCloskey-Mueller Satisfaction Scale; CI: Confidence Interval; OR: Odds Ratios

## Competing interests

The authors declare that they have no competing interests.

## Authors' contributions

FE was the principal investigator on this study and contributed to the conception, design, as well as analysis and interpretation of results. HD was involved in conceptualizing and implementing the data analysis plan as well as overseeing drafting of research results. ND was a co-investigator on this study and also contributed to the conception and design as well as the writing of this manuscript. DJ made substantial contributions to project management, including data collection and analysis as well as in drafting the manuscript. GM was a co-investigator on this study and also contributed to the data collection plan as well as revising the final manuscript. Authors read and approved the final manuscript.

## Pre-publication history

The pre-publication history for this paper can be accessed here:



## Supplementary Material

Additional File 1 **Questionnaire. This file includes the survey used in this study.** Click here for file

Additional File 2 **Additional File 1: Association between intent to leave and nurses’ characteristics (N=1793)**. The data provided in the table summarizes the outcomes of a cross-tabulation of the variable on intent to leave against nurses' characteristics. Click here for file
