# Supplementary material for: A national cross-sectional study on nurses' intent to leave and job satisfaction in Lebanon: implications for policy and practice
Source: BMC Nurs. 2009 Mar 12;8:3. doi: 10.1186/1472-6955-8-3 (PMC2667438; doi:10.1186/1472-6955-8-3)
Supplement: Additional File 1 — Questionnaire. This file includes the survey used in this study. [file 1472-6955-8-3-S1.doc]

**Questionnaire for**

**A National Cross-sectional Study on Nurses’ Intent to Leave and Job Satisfaction in Lebanon: Implications for Policy and Practice**

**A- Demographic Characteristics**

1. **Gender**
   1. Female
   2. Male
2. **Age?**
   1. Below 30 years
   2. Between 30 and 45 years
   3. Between 46 and 55 years
   4. Over 55 years
3. **Marital Status**
   1. Never married
   2. Ever married (includes married, divorced or widowed)
4. **What is your highest degree?**
   1. Diploma in Nursing
   2. Baccalaureate Technical (BT)
   3. Technique Superior (TS)
   4. License Technique (LT)
   5. Bachelor of Sciences
   6. Masters degree
   7. Other: ____________________________
5. **Where do you reside?**
   1. Beirut
   2. Mount Lebanon
   3. North
   4. Bekaa
   5. South
   6. Nabatieh

************************************************************

**B- Questions about intent to leave and plans after leaving**

1. **Thinking about the next 1-3 years, do you…**
   1. Intend to stay in your current job (if you choose this option, please skip to question 8)
   2. Intend to leave your current job
2. **If you are thinking of leaving your current job, please circle the best 3 options that describe your plans after leaving.**
   1. Move to another hospital in Lebanon
   2. Leave country
   3. Change nursing profession
   4. Continue education, specify degree________________
   5. Take care of children or other dependents (parents)
   6. Other, specify___________________________________
3. **In your opinion, finding another job in nursing would be…**
   1. Easy
   2. Difficult
4. **Given the opportunity to start all over, would choose nursing as a profession?**
   1. Yes
   2. No

************************************************************

**MCLOSKEY / MUELLER SATISFACTION SCALE (MMSS)** (© 1989)

**HOW SATISFIED ARE YOU WITH THE FOLLOWING ASPECTS OF YOUR CURRENT JOB?**

|  | | **Very Dissatisfied** | **Dissatisfied** | **Satisfied** | **Very Satisfied** | **Not Applicable** |
| --- | --- | --- | --- | --- | --- | --- |
| 1 | Salary | 1 | 2 | 3 | 4 | 8 |
| 2 | Vacation | 1 | 2 | 3 | 4 | 8 |
| 3 | Benefit package (insurance, retirement) | 1 | 2 | 3 | 4 | 8 |
| 4 | Hours that you work | 1 | 2 | 3 | 4 | 8 |
| 5 | Flexibility in scheduling your hours | 1 | 2 | 3 | 4 | 8 |
| 6 | Opportunity to work straight days | 1 | 2 | 3 | 4 | 8 |
| 7 | Opportunity for part-time work | 1 | 2 | 3 | 4 | 8 |
| 8 | Weekends off per month | 1 | 2 | 3 | 4 | 8 |
| 9 | Flexibility in scheduling your weekends | 1 | 2 | 3 | 4 | 8 |
| 10 | Compensation for working weekends | 1 | 2 | 3 | 4 | 8 |
| 11 | Maternity leave time | 1 | 2 | 3 | 4 | 8 |
| 12 | Child care facilities for employees’ children in the hospital | 1 | 2 | 3 | 4 | 8 |
| 13 | Your head nurse/nurse manager | 1 | 2 | 3 | 4 | 8 |
| 14 | Your nursing peers | 1 | 2 | 3 | 4 | 8 |
| 15 | The physicians you work with | 1 | 2 | 3 | 4 | 8 |
| 16 | The delivery of care method used on your unit (e.g., functional, team, primary, modular care, patient-centered care) | 1 | 2 | 3 | 4 | 8 |
| 17 | Opportunities for social contact at work | 1 | 2 | 3 | 4 | 8 |
| 18 | Opportunities for social contact with your colleagues after work | 1 | 2 | 3 | 4 | 8 |
| 19 | Opportunities to interact professionally with other disciplines | 1 | 2 | 3 | 4 | 8 |
| 20 | Opportunities to interact with faculty | 1 | 2 | 3 | 4 | 8 |
| 21 | Opportunities to belong to department and institutional committees | 1 | 2 | 3 | 4 | 8 |
| 22 | Control over what goes on in your work setting | 1 | 2 | 3 | 4 | 8 |
| 23 | Opportunities for career advancement | 1 | 2 | 3 | 4 | 8 |
| 24 | Recognition of your work from superiors | 1 | 2 | 3 | 4 | 8 |
| 25 | Recognition of your work from peers | 1 | 2 | 3 | 4 | 8 |
| 26 | Amount of encouragement and positive feedback | 1 | 2 | 3 | 4 | 8 |
| 27 | Opportunities to participate in nursing research | 1 | 2 | 3 | 4 | 8 |
| 28 | Opportunities to write and publish | 1 | 2 | 3 | 4 | 8 |
| 29 | Your amount of responsibility | 1 | 2 | 3 | 4 | 8 |
| 30 | Your control of work conditions | 1 | 2 | 3 | 4 | 8 |
| 31 | Your participation in organizational decision making | 1 | 2 | 3 | 4 | 8 |
