# Supplementary material for: A national cross-sectional study on nurses' intent to leave and job satisfaction in Lebanon: implications for policy and practice
Source: BMC Nurs. 2009 Mar 12;8:3. doi: 10.1186/1472-6955-8-3 (PMC2667438; doi:10.1186/1472-6955-8-3)
Supplement: Additional File 2 — Additional File 1: Association between intent to leave and nurses’ characteristics (N=1793). The data provided in the table summarizes the outcomes of a cross-tabulation of the variable on intent to leave against nurses' characteristics. [file 1472-6955-8-3-S2.doc]

**Additional File 1: Association between intent to leave and nurses’ characteristics** (N=1793)

|  |  | | **Stay** | **Intent to leave Job** | | | |  |
| --- | --- | --- | --- | --- | --- | --- | --- | --- |
|  |  | |  | **Total** | **P-Value (1)** | **Leave Hospital** | **Leave Country** | **P-Value (2)** |
|  |  | | **N (%)** | **N (%)** |  | **N (%)** | **N (%)** |  |
| **Age** | | |  |  |  |  |  |  |
|  | < 30 years | | 311 (55.5) | 807 (69.2) | **<0.001** | 468 (63.3) | 339 (79.2) | **<0.001** |
|  | 30 to 45 years | | 213 (38.0) | 333 (28.5) |  | 249 (33.7) | 84 (19.6) |  |
|  | 46 to 55 years | | 35 (6.2) | 22 (1.9) |  | 18 (2.4) | 4 (0.9) |  |
|  | > 55 years | | 1 (0.2) | 5 (0.4) |  | 4 (0.5) | 1 (0.2) |  |
| **Gender** | | |  |  |  |  |  |  |
|  | Female | | 481 (84.1) | 949 (79.9) | **0.037** | 657 (87.3) | 292 (67.3) | **<0.001** |
|  | Male | | 91 (15.9) | 238 (20.1) |  | 96 (12.7) | 142 (32.7) |  |
| **Marital Status** | | |  |  |  |  |  |  |
|  | Never married | | 306 (53.5) | 701 (59.5) | **0.017** | 390 (52.3) | 311 (72.0) | **<0.001** |
|  | Ever Married | | 266 (46.5) | 477 (40.5) |  | 356 (47.7) | 121 (28.0) |  |
| **Region** | | |  |  |  |  |  |  |
|  | Beirut | | 80 (19.4) | 219 (24.3) | **0.017** | 126 (22.5) | 93 (27.3) | **0.034** |
|  | Mount Lebanon | | 180 (43.7) | 304 (33.8) |  | 186 (33.3) | 118 (34.6) |  |
|  | North | | 49 (11.9) | 112 (12.4) |  | 76 (13.6) | 36 (10.6) |  |
|  | Bekaa | | 49 (11.9) | 109 (12.1) |  | 74 (13.2) | 35 (10.3) |  |
|  | South | | 35 (8.5) | 104 (11.6) |  | 63 (11.3) | 41 (12.0) |  |
|  | Nabatieh | | 19 (4.6) | 52 (5.8) |  | 34 (6.1) | 18 (5.3) |  |
| **Degree Type** | | |  |  |  |  |  |  |
|  | University | | 194 (34.1) | 426 (36.2) | 0.397 | 233 (31.3) | 193 (44.5) | **<0.001** |
|  | |  |  |  |  |  |  |  |
|  | | BSN | 182 (31.8) | 389 (32.9) |  | 215 (28.7) | 174 (40.0) |  |
|  | | Masters | 12 (2.1) | 37 (3.1) |  | 18 (2.4) | 19 (4.4) |  |
|  |  | |  |  |  |  |  |  |
|  | Technical | | 375 (65.9) | 752 (63.8) |  | 511 (68.7) | 241 (55.5) |  |
|  | |  |  |  |  |  |  |  |
|  | | Diploma | 67 (11.7) | 144 (12.2) |  | 84 (11.2) | 60 (13.8) |  |
|  | | BT | 96 (16.8) | 153 (12.9) |  | 123 (16.4) | 30 (6.9) |  |
|  | | TS | 132 (23.0) | 254 (21.5) |  | 178 (23.8) | 76 (17.5) |  |
|  | | LT | 67 (11.7) | 173 (14.6) |  | 107 (14.3) | 66 (15.2) |  |
|  | | Others | 17 (3.0) | 34 (2.9) |  | 24 (3.2) | 10 (2.3) |  |
| **Finding another job in nursing would be** | | |  |  |  |  |  |  |
|  | Easy | | 294 (53.6) | 688 (59.1) | **0.030** | 416 (57.1) | 272 (62.5) | **0.018** |
|  | Difficult | | 255 (46.4) | 476 (40.9) |  | 313 (42.9) | 163 (37.5) |  |
| **Given the opportunity to start all over, would nurses intending to leave choose nursing as a profession?** | | |  |  |  |  |  |  |
|  | Yes | | 327 (58.4) | 431 (37.1) | **<0.001** | 256 (34.8) | 175 (40.9) | **<0.001** |
|  | No | | 233 (41.6) | 732 (62.9) |  | 479 (65.2) | 253 (59.1) |  |

1. P-Value to explore the difference between nurses who intend to stay and those who intend to leave job
2. P-Value to explore the difference between nurses who intend to stay against those who want to leave the hospital or leave the country
